# Microstructure, Corrosion Resistance and Mechanical Properties of Dissimilar Welded Austenitic, Super-Austenitic and Duplex Stainless Steels

**DOI:** 10.3390/ma19214583

**Published:** 2026-10-29

**Authors:** Isidoros Iakovidis, Stavros Chionopoulos, Ioannis Rachoutis

**Affiliations:** Department of Naval Architecture, University of West Attica, 12243 Athens, Greece; schionopoulos@uniwa.gr (S.C.); mscna2208@uniwa.gr (I.R.)

**Keywords:** dissimilar stainless-steel welding, 254 SMO, 310S, 2205 duplex, ERNiCrMo-3, microstructure, tensile properties, pitting corrosion

## Abstract

**Highlights:**

- ERNiCrMo-3 successfully joined 254 SMO, 310S, and 2205 in three dissimilar combinations.
- Base-metal-dependent dilution influenced weld-metal chemistry and microstructural heterogeneity.
- 254 SMO–310S and 254 SMO–2205 fractured outside the weld zone under tensile loading.
- All joints showed limited mass loss, with no preferential localized attack of the weld metal.

**Abstract:**

This study aimed to comparatively evaluate the microstructural characteristics, mechanical properties, and localized corrosion resistance of three dissimilar stainless-steel joints: 254 SMO^®^ (UNS S31254)–310S (UNS S31008), 254 SMO^®^ (UNS S31254)–2205 (UNS S32205), and 310S (UNS S31008)–2205 (UNS S32205). The joints were produced by gas tungsten arc welding (GTAW) using ERNiCrMo-3 filler metal and were investigated through macrostructural examination, SEM–EDS analysis, microhardness measurements, transverse tensile testing, and ASTM G48 corrosion testing. All three combinations produced fully penetrated welds without visible macroscopic discontinuities. SEM–EDS revealed Fe–Ni gradients across the fusion boundaries, consistent with local dilution of the Ni-based filler metal, together with Nb–Mo-rich interdendritic constituents and discrete Ti–Nb-rich particles in the weld metal. The 254 SMO–310S joint exhibited the highest tensile strength and elongation (776 MPa and 43.6%), followed by 254 SMO–2205 (742 MPa and 27.7%), with both fracturing outside the weld zone. The 310S–2205 joint showed lower strength and ductility (578 MPa and 12.3%) and fractured within the welded region. ASTM G48 testing showed low mass losses and no pronounced preferential attack of the weld metal or heat-affected zones. ERNiCrMo-3 produced sound dissimilar joints in all three material combinations, although their microstructural, mechanical, and corrosion responses differed with the adjoining base metals.

## 1. Introduction

Austenitic (ASS), super-austenitic (SASS), and duplex (DSS) stainless steels are widely employed in shipbuilding, marine engineering, offshore, chemical, petrochemical, energy, and desalination industries owing to their favorable combination of mechanical strength, toughness, corrosion resistance, durability, and weldability [1,2,3,4]. Conventional ASS is extensively used for piping, tanks, fittings, equipment, and structural components, whereas SASS and DSS are increasingly selected for demanding service conditions involving seawater and other aggressive environments. The term “super-austenitic” refers to highly alloyed austenitic stainless steels characterized by elevated contents of corrosion-resistant alloying elements, particularly Cr, Mo, and N, together with sufficient Ni to stabilize the austenitic structure. Compared with conventional austenitic grades, these alloys exhibit substantially enhanced resistance to localized corrosion, especially pitting and crevice corrosion in chloride-containing environments. SASS provide exceptional resistance to localized corrosion, particularly pitting and crevice corrosion, as well as chloride-induced stress-corrosion cracking [5,6,7]. DSS, characterized by an approximately balanced ferritic–austenitic microstructure, combine high mechanical strength with good resistance to general and localized corrosion and enhanced resistance to chloride-induced stress-corrosion cracking compared with conventional ASS [2,8,9]. These characteristics make SASS and DSS particularly attractive for applications such as chemical and product tankers, offshore structures, seawater and process piping, desalination plants, ballast-water systems, and marine exhaust-gas cleaning equipment [2,10]. However, the fabrication and welding of these alloys require careful control because the thermal cycles associated with welding can substantially modify their microstructure and, consequently, their mechanical and corrosion performance.

The thermal history during welding affects phase transformations [2,8], solidification behavior, and precipitation reactions through the peak temperature, heating and cooling rates, and time spent within critical temperature ranges [3,9]. Inappropriate welding conditions may promote the formation of detrimental secondary phases, such as sigma (σ) and chi (χ) phases and chromium nitrides, and may disturb the ferrite–austenite balance and the formation of secondary austenite [2,3,8,9]. In highly alloyed stainless steels, precipitation reactions may also be accompanied by localized depletion of essential alloying elements, particularly Cr and Mo, from the surrounding matrix [4,9]. Such microstructural and compositional changes can impair mechanical performance and corrosion resistance [2,3,4,8,9] and increase susceptibility to intergranular and pitting corrosion [4,6,7], chloride-induced stress-corrosion cracking [5], and, under unfavorable solidification conditions, cracking during or after welding [3,9].

These effects become more complex when dissimilar stainless steels are joined because the base materials differ substantially in alloy chemistry, phase stability, and solidification behavior. The welding process must therefore accommodate the requirements of each alloy simultaneously. For the austenitic side, preservation of corrosion resistance, avoidance of sensitization, and adequate resistance to solidification cracking are important. For the duplex side, maintaining the appropriate phase constitution while limiting detrimental transformations is essential. For the super-austenitic side, preservation of the highly alloyed Cr-Mo-N composition responsible for its localized-corrosion resistance is particularly important.

In dissimilar joints, dilution and welding thermal history jointly influence the weld-metal microstructure. Dilution refers to the contribution of molten base-metal material to the final weld-metal composition through mixing with the deposited filler metal. In dissimilar welding, the relative contributions of the two base metals may differ depending on the local fusion geometry and welding conditions, resulting in compositional variations within the fusion zone. These effects are particularly relevant when using Ni-based filler metals, such as ERNiCrMo-3, to join stainless steels with substantially different Fe, Ni, Cr, and Mo contents. Local changes in chemical composition can influence solidification behavior, elemental segregation, and the formation of secondary constituents, particularly in interdendritic regions. In parallel, the welding thermal cycle governs heating and cooling rates, phase transformations, and precipitation reactions. Consequently, the resulting weld metal should be regarded as a compositionally and microstructurally heterogeneous region rather than a homogeneous mixture of the filler and base metals. Filler-metal selection should therefore consider the chemistry of both base metals and the expected dilution rather than solely nominal chemical matching. Dissimilar combinations such as 316L/2205, 316L/2507, 254 SMO/2507, and AL6XN/2205 have been successfully welded, although each combination presents specific metallurgical challenges related to the different base-metal compositions and microstructures [3,11,12,13,14].

The effects of dissimilar welding extend beyond the fusion zone, into the heat-affected zones (HAZ). Investigations of dissimilar ASS/SDSS welds have shown that the SDSS HAZ may undergo partial ferritization, whereas the ASS HAZ can exhibit recrystallization, grain growth, and changes in crystallographic texture [15,16]. Such transformations are important because variations in the duplex phase constitution can influence hardness, tensile strength, toughness, and corrosion behavior. Changes in heat input and cooling conditions can therefore affect the local properties of both the weld metal and HAZ [17,18].

Filler-metal chemistry provides an additional means of influencing the composition and solidification behavior of the weld metal. Its influence is particularly important in dissimilar joints because the filler interacts with two base metals having different alloy chemistry. Ramkumar et al. reported that the use of a super-duplex filler in 316L ASS/S32750 SDSS joints produced a relatively balanced ferritic–austenitic weld microstructure and superior impact toughness, whereas the nickel-based ERNiCrMo-3 filler promoted secondary phases enriched in Ni, Nb, and Mo [19]. Similarly, comparisons of austenitic, super-austenitic, and super-duplex fillers for 316L ASS/S32205 DSS joints demonstrated that filler chemistry strongly affected phase constitution and the resulting balance between strength, hardness, toughness, and corrosion resistance [20].

During solidification, segregation of Cr, Mo, Nb, and other alloying elements may generate local compositional heterogeneity and promote the formation of intermetallic, carbide, and nitride phases. In 2205 DSS/316L ASS joints, for example, σ phase formation associated with an unsuitable filler metal was accompanied by chromium depletion and deterioration of corrosion resistance, whereas an alternative filler selection resulted in a more favorable weld microstructure and improved electrochemical performance [11]. Recent electrochemical impedance spectroscopy investigations have likewise demonstrated that passive-film stability in 316L/2205 dissimilar welds depends on welding conditions, filler-metal selection, and the microstructure developed within the joint [12].

This issue is particularly relevant to SASS/DSS combinations, in which the highly alloyed composition of SASS contrasts with the two-phase ferritic–austenitic structure of DSS. Studies of 904L SASS/S32750 SDSS joints have reported microsegregation of Mo-, Cr-, and W-rich constituents in the fusion zone and grain coarsening in the SDSS HAZ, with the resulting microstructure influencing both mechanical properties and corrosion behavior [13]. Similarly, laser-welded 2507 SDSS/904L SASS joints demonstrated that heat input and post-weld heat treatment significantly affected weld-metal morphology, phase fractions, grain structure, strength, hardness, ductility, and impact toughness [21]. Zhou and Löthman further demonstrated that filler-metal composition significantly affected weld-metal microstructure, secondary-phase formation, and corrosion behavior in dissimilar super-duplex/super-austenitic stainless-steel welds [22].

The significance of ERNiCrMo-3 filler chemistry is further illustrated by investigations of AL6XN SASS/2205 DSS joints, where its use was associated with significant ferritization and changes in the phase constitution on the duplex side, together with elemental segregation and the formation of Nb- and Mo-rich precipitates, including Laves phases and niobium carbides [14]. These observations are particularly relevant to the present study because the relatively high Ni, Cr, Mo, and Nb contents of ERNiCrMo-3 can interact with the substantially different compositions of the stainless-steel base metals. Related investigations of DSS/Ni-alloy dissimilar welding have likewise highlighted the effects of dilution, chemical heterogeneity, intermetallic-phase formation, and filler-metal selection on weld performance [19,23,24,25].

The corrosion response of dissimilar stainless-steel joints is closely related to the local microstructural heterogeneity produced by these metallurgical interactions. Differences in phase constitution, elemental redistribution, and secondary-phase precipitation can establish electrochemical heterogeneity and create preferential sites for localized corrosion. Dissimilar 904L/super-duplex stainless-steel welds have been reported to exhibit susceptibility to microbiologically influenced corrosion [13]. Furthermore, investigations of 310S/duplex stainless-steel dissimilar welds have demonstrated that the duplex-side HAZ may become particularly susceptible to stress-corrosion cracking as a consequence of welding-induced microstructural changes and secondary-phase precipitation [26].

Previous studies of ASS/DSS and SASS/DSS dissimilar welding have mainly focused on individual material combinations. Studies have addressed conventional ASS/SDSS systems [11,12,15,16,17,18,19,20], SASS/SDSS combinations [13,21,22], and SASS/DSS systems [14]. However, direct comparisons of 254 SMO ^®^ (UNS S31254)–310S (UNS S31008), 254 SMO ^®^ (UNS S31254)–2205 (UNS S32205), and 310S (UNS S31008)–2205 (UNS S32205) joints produced with the same filler metal and under comparable welding conditions are limited. Such a comparison allows the influence of base-metal-dependent dilution on weld-metal chemistry, microstructure, mechanical response, and localized corrosion behavior to be examined using the same ERNiCrMo-3 filler metal.

The present study compares 254 SMO–310S, 254 SMO–2205, and 310S–2205 dissimilar joints produced by gas tungsten arc welding (GTAW) using ERNiCrMo-3 filler metal. The joints were evaluated in terms of macrostructure, weld-metal and HAZ microstructure, elemental distribution across the fusion boundaries, microhardness, transverse tensile properties, and localized corrosion behavior. Particular attention was given to the relationship between base-metal dilution, weld-metal heterogeneity, and the resulting mechanical and corrosion response of each material combination.

## 2. Materials and Methods

### 2.1. Materials and Weld Fabrication

**Figure 1:**
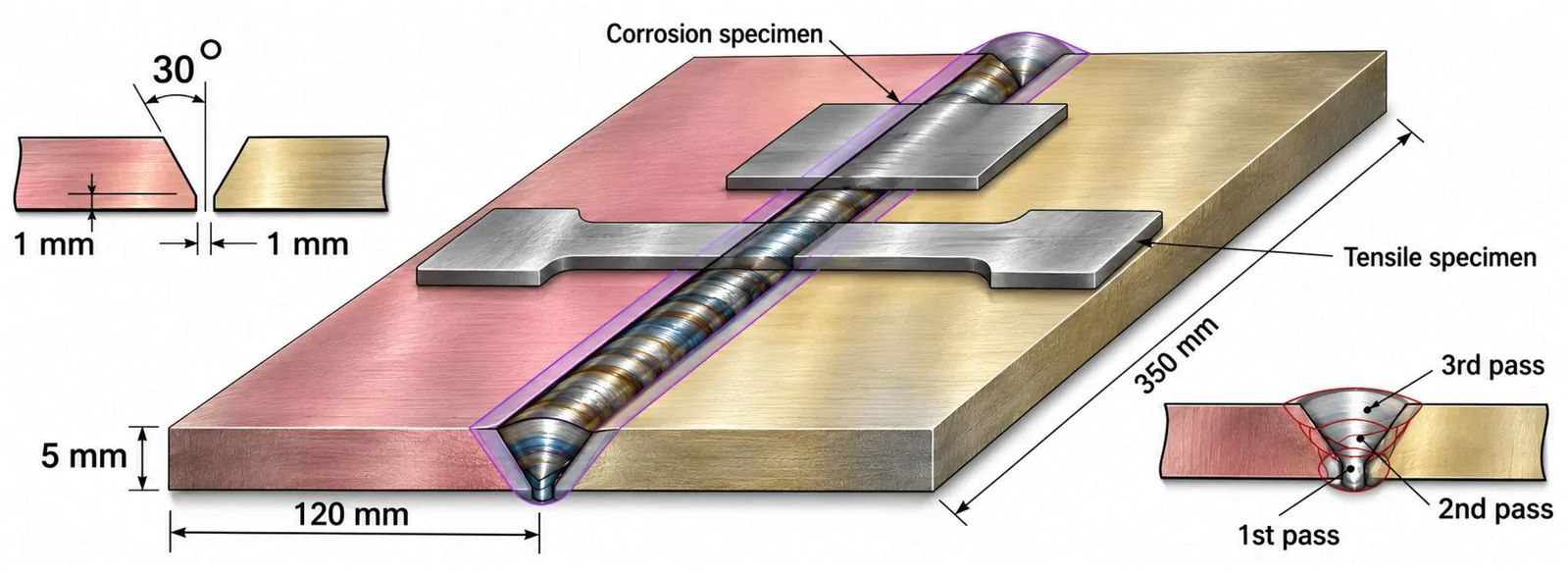
Schematic representation of the dissimilar welded assembly, illustrating the joint geometry and dimensions, three-pass GTAW sequence, and locations of the tensile and corrosion test specimens.

Plates of 310S austenitic stainless steel (UNS S31008), 254 SMO super-austenitic stainless steel (UNS S31254), and 2205 duplex stainless steel (UNS S32205) were used to fabricate three separate dissimilar butt-joint combinations: 254 SMO–310S, 254 SMO–2205, and 310S–2205. Each base-metal plate had nominal dimensions of 350 mm × 120 mm × 5 mm (length × width × thickness) prior to welding. For each dissimilar material combination, two plates, measuring 350 × 120 × 5 mm each, were joined by GTAW along their 350 mm edges, producing a welded assembly with nominal overall dimensions of 350 × 240 × 5 mm (Figure 1). The nominal chemical compositions of the base metals and filler metal are presented in Table 1. One welded assembly was produced for each dissimilar material combination. One transverse tensile specimen and one corrosion specimen were tested per combination.

Before welding, the joint areas were ground using tools dedicated to stainless steel. The plate edges were machined to form a single-V groove with a 30° bevel on each edge, a 1 mm root face, and a 1 mm root gap (Figure 1).

The welded assemblies were produced without external mechanical restraints, allowing free deformation during welding and cooling and avoiding additional stresses associated with mechanical clamping. The three dissimilar joints were welded in three passes by manual gas tungsten arc welding (GTAW/TIG; process 141 according to EN ISO 4063 [27]), in the PA (flat) position according to EN ISO 6947 [28]. Welding was performed using direct current electrode negative (DCEN) polarity and a WT20 thoriated tungsten electrode (2 wt.% ThO_2_), 2.4 mm in diameter, according to EN ISO 6848 [29]. ERNiCrMo-3 filler metal (AWS A5.14/A5.14M; EN ISO 18274 [30]: S Ni 6625), with a wire diameter of 2.0 mm, was manually added during welding. High-purity argon (Ar 5.0, 99.999%), classified as I1 according to EN ISO 14175 [31], was used as shielding gas at a flow rate of 12 L/min. No backing gas was used. The initial plate temperature was approximately 20 °C, and the maximum interpass temperature was 110 °C. Joint preparation and cleaning were performed by brushing and/or grinding using tools dedicated to stainless steel. The welding parameters are summarized in Table 2. The heat input for each welding pass was calculated from the recorded welding current, arc voltage, and travel speed using the relationship H = ηUI/v, where η is the arc thermal efficiency, U is the arc voltage, I is the welding current, and v is the travel speed. A thermal efficiency factor of 0.60 was adopted for the GTAW process. The calculated heat input values ranged from 1.08 to 1.30 kJ/mm. These variations resulted from the actual welding conditions recorded during manual GTAW and were not intentionally introduced as an experimental variable.

### 2.2. Microstructural and Microhardness Examination

Transverse sections were cut from each welded joint to include the weld metal, both fusion boundaries, the heat-affected zones (HAZs), and the adjacent base metals. The sections were mounted in conductive resin, ground, and polished. Electrolytic etching was performed in a 10% aqueous oxalic acid solution using a Struers electrolytic preparation unit (Struers ApS, Ballerup, Denmark) at an applied voltage of 3–5 V; the etching time was adjusted according to the material response.

The weld cross-sections were examined using an Olympus SZX10 (Olympus Corporation, Tokyo, Japan) stereomicroscope equipped with a digital camera and image-analysis software. Microstructural examination and local elemental analysis were subsequently performed using a Thermo Scientific Phenom XL scanning electron microscope (SEM) equipped with energy-dispersive X-ray spectroscopy (EDS) (Thermo Fisher Scientific, Eindhoven, The Netherlands).

Vickers microhardness measurements were performed using a QATM Qness 60 A + EVO tester (ATM Qness GmbH, Mammelzen, Germany) under a test force of 0.5 kgf (HV0.5). Indentations were arranged in line profiles across the welded joints and in two-dimensional grids for hardness mapping, covering the base metals, HAZs, and weld metal, with a spacing of 500 μm between adjacent indentations. Two-dimensional hardness maps were generated automatically using the Qness 60 software, with color interpolation between the measured indentation points. The corresponding linear hardness profiles present the measured hardness values as a function of distance.

### 2.3. Tensile Testing

Flat transverse tensile specimens were extracted from the central region of each welded assembly, perpendicular to the welding direction, with the weld positioned at the center of the parallel section (Figure 1). The specimens were produced by mechanical cutting and CNC machining using conventional cutting tools. Weld reinforcement was removed from both surfaces to obtain a uniform specimen thickness. Specimen geometry and preparation followed EN ISO 4136:2022 [32]. For the 5 mm plate thickness, the nominal width of the parallel section was 25 mm, the gripping-section width was 37 mm, and the transition radius was at least 25 mm. The nominal cross-sectional area of the parallel section was therefore 125 mm^2^. One transverse tensile specimen was tested for each dissimilar material combination. Tensile testing was performed at room temperature in accordance with ISO 6892-1 [33] using a GALDABINI Sun 60 universal testing machine (Galdabini S.p.A., Cardano al Campo, Varese, Italy) with a maximum load capacity of 600 kN. Engineering stress–strain curves were used to determine the ultimate tensile strength (UTS), 0.2% proof strength (Rp0.2), and elongation of the tested specimens. The initial gauge length (L_0_) was 63.2 mm, corresponding to the proportional specimen relationship L_0_ = 5.65√S_0_. Strain was measured using an extensometer, and tensile testing was performed at a nominal strain rate of 2.5 × 10^−4^ s^−1^ in accordance with ISO 6892-1 [33].

### 2.4. Pitting Corrosion Testing

The resistance of the dissimilar welded joints to localized corrosion was evaluated using the ferric chloride pitting corrosion test in accordance with ASTM G48, Method A [34]. The test solution was prepared by dissolving 100 g of FeCl_3_·6H_2_O in 900 mL of reagent water, corresponding to approximately 6 wt.% FeCl_3_. Testing was conducted using 600 mL of solution at 22 ± 2 °C. Specimen surface preparation, edge finishing, and post-exposure cleaning followed ASTM G48, Method A. Rectangular corrosion specimens measuring approximately 50 × 25 × 5 mm were extracted from each welded assembly at approximately two-thirds of the weld length (2L/3), measured along the welding direction (Figure 1). The specimens were oriented transversely to the weld axis, with the welded joint approximately centered within each specimen. Thus, each specimen included weld metal, both fusion boundaries, the adjacent heat-affected zones, and portions of both base metals. One corrosion specimen was tested for each dissimilar material combination. Since the plates were joined in a butt-joint configuration, the nominal specimen thickness remained 5 mm, corresponding to the thickness of the individual plates. The specimens were immersed in ferric chloride solution for 72 h at room temperature, rinsed, cleaned, dried, and weighed using an analytical balance with a resolution of ±0.0001 g to determine mass loss, while the evaluation of pitting corrosion was based on the examination of the exposed specimen surfaces in accordance with ASTM G48 Method A.

## 3. Results

### 3.1. Weld Macrostructure and Heat-Affected Zones

**Figure 2:**
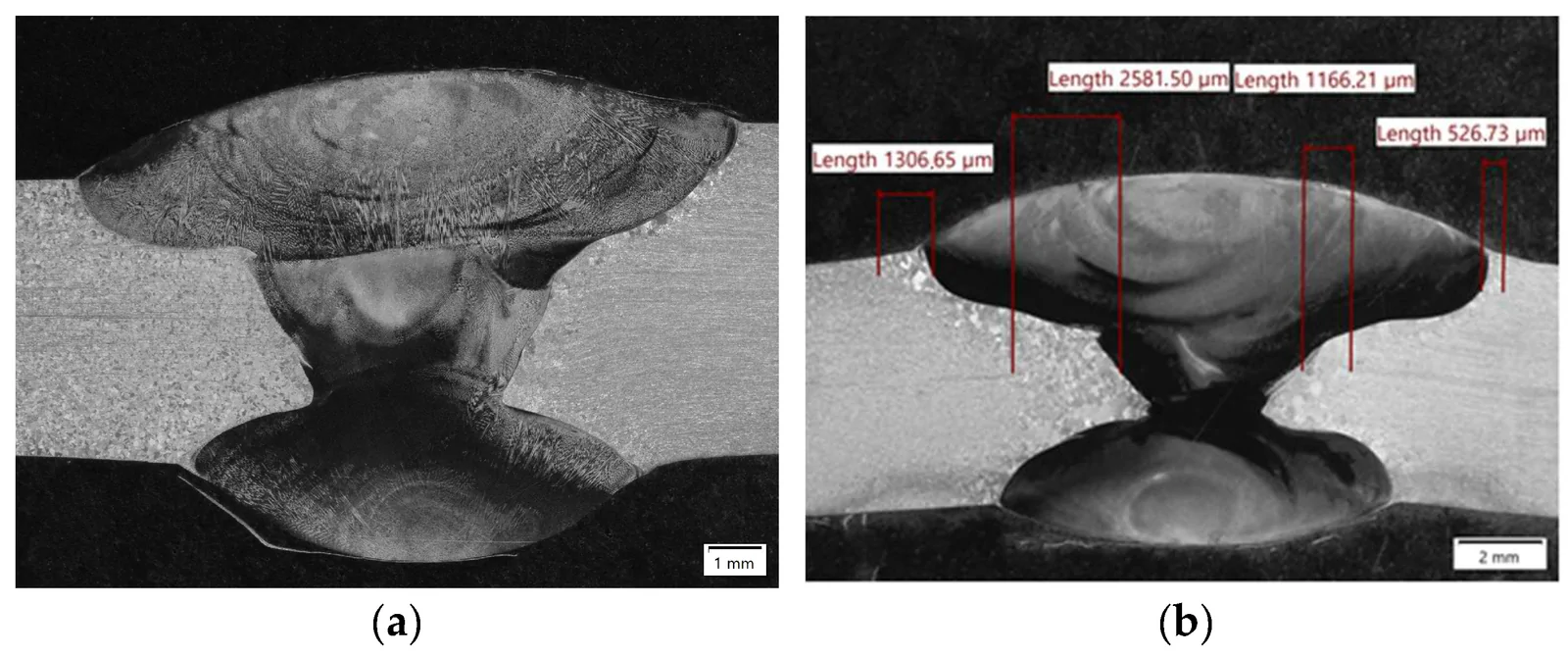
Stereomicrographs of the 254 SMO–310S weld cross-section: (**a**) overall weld profile and successive passes; (**b**) heat-affected zone widths measured from the fusion line into the base metal near the weld cap and mid-thickness region on the 254 SMO and 310S sides using calibrated image analysis. The 254 SMO is on the left and 310S is on the right.

**Figure 3:**
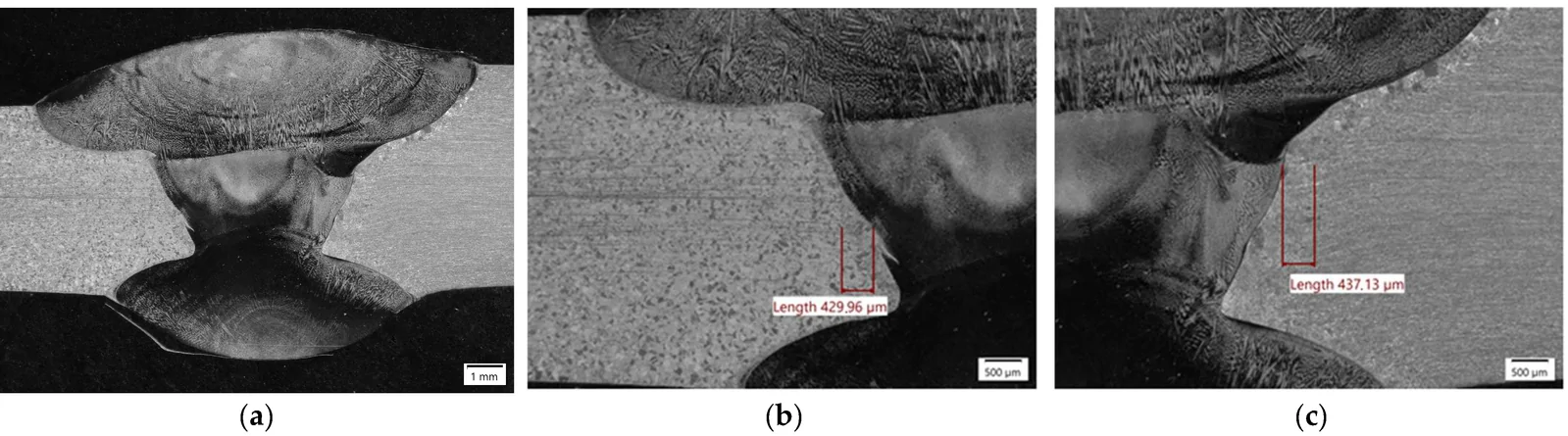
Stereomicrographs of the 254 SMO–2205 weld cross-section: (**a**) overall weld profile and successive passes (the 254 SMO base metal is shown on the left, while the 2205 base metal is shown on the right); (**b**) fusion boundary and local HAZ width on the 254 SMO side (0.43 mm); (**c**) fusion boundary and local HAZ width on the 2205 side (0.44 mm).

**Figure 4:**
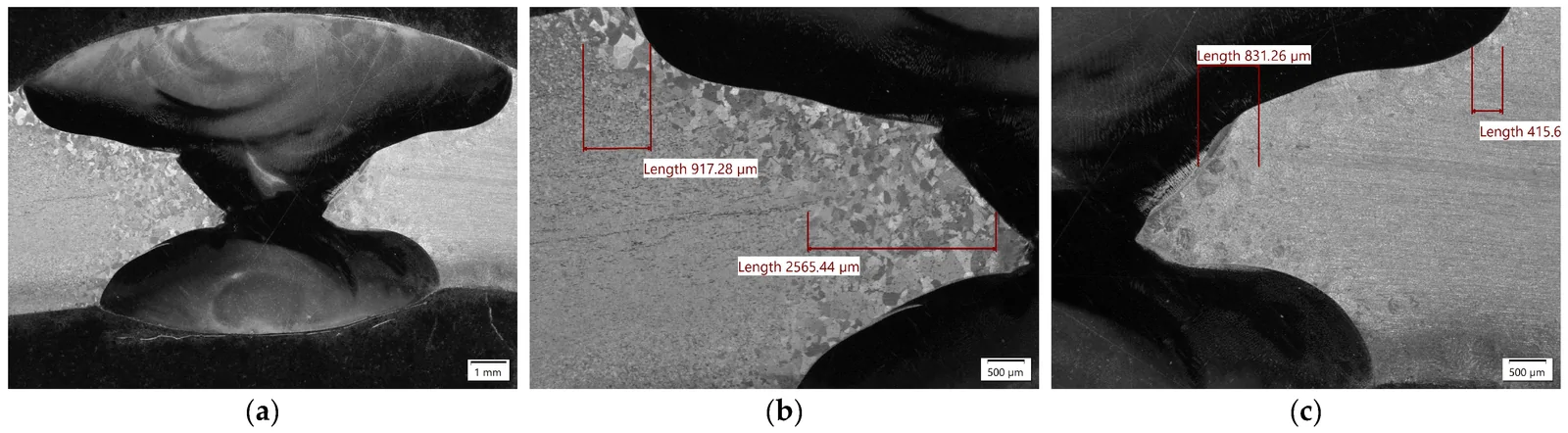
Stereomicrographs of the 310S–2205 weld cross-section: (**a**) overall weld profile and successive passes (the 310S base metal is shown on the left, while the 2205 base metal is shown on the right); (**b**) local HAZ widths on the 310S side (0.92 and 2.56 mm); (**c**) local HAZ widths on the 2205 side (0.83 and 0.42 mm).

The stereomicrographs of the 254 SMO–310S joint show a continuous, fully penetrated weld in the examined cross-section, with visible face and root reinforcement (Figure 2a). The successive welding passes can be distinguished in the etched cross-section. No macroscopic cracks, pores, incomplete penetration, or indications of lack of fusion were observed in the examined cross-section at the magnification employed. The apparent HAZ widths were assessed at different locations along the fusion boundaries, with representative local measurements presented in Figure 2, Figure 3 and Figure 4.

The weld metal exhibits predominantly columnar solidification features extending from the fusion boundaries toward the interior of each pass. Their orientation changes with the local weld-pool geometry and from one pass to another. In the wider upper pass, the central region shows less strongly directional features, with a more equiaxed appearance. The apparent width of the etched HAZ varied along the fusion boundary (Figure 2b). On the 254 SMO side, the measured widths were 1.32 mm near the weld cap and 2.58 mm near the mid-thickness region, corresponding to a mean of 1.94 mm for the two measured locations. The corresponding widths on the 310S side were 0.53 mm and 1.17 mm, giving a mean of 0.85 mm for the two measured locations. The apparent etched HAZ was wider on the 254 SMO side at both measured locations.

The 254 SMO–2205 joint shows a fully penetrated weld with distinct face and root reinforcement (Figure 3a). The boundaries between successive passes and changes in the orientation of the columnar solidification features are visible across the weld metal. No macroscopic pores, cracks, or clear indications of incomplete fusion were observed in the examined cross-section. Along the visible fusion boundaries, the apparent width of the etched HAZ showed relatively little variation from the weld cap toward the mid-thickness region. The local HAZ widths measured by calibrated image analysis were 0.43 mm on the 254 SMO side (Figure 3b) and 0.44 mm on the 2205 side (Figure 3c).

The 310S–2205 joint shows full penetration, with visible face and root reinforcement and distinguishable weld passes (Figure 4a). No macroscopic pores, cracks, or clear indications of incomplete fusion were observed in the examined cross-section. The apparent width of the etched HAZ varied along both fusion boundaries. On the 310S side, the HAZ widths measured near the weld cap and near the mid-thickness region were 0.92 mm and 2.56 mm, respectively, corresponding to a mean of 1.74 mm for the two measured locations (Figure 4b). On the 2205 side, the corresponding values were 0.83 mm and 0.42 mm, giving a mean of 0.62 mm (Figure 4c). The apparent width of the etched HAZ was wider on the 310S side at both examined locations.

### 3.2. SEM–EDS Microstructural Analysis

#### 3.2.1. Base-Metal Microstructures

**Figure 5:**
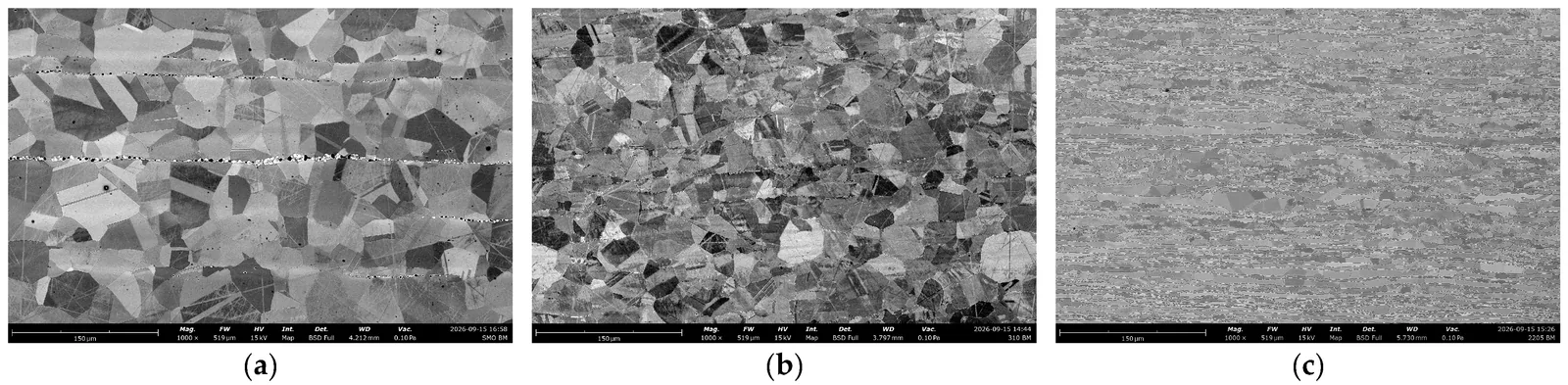
SEM micrographs of the base metals: (**a**) 254 SMO, (**b**) 310S, and (**c**) 2205 duplex stainless steel.

The 254 SMO and 310S base metals exhibit equiaxed austenitic grains with visible annealing twins (Figure 5a,b). In the examined fields, the grains appear generally larger in 254 SMO than in 310S. The 2205 base metal exhibits a duplex ferrite–austenite microstructure, with elongated, approximately parallel phase bands following the plate rolling direction (Figure 5c).

#### 3.2.2. Heat-Affected Zones

**Figure 6:**
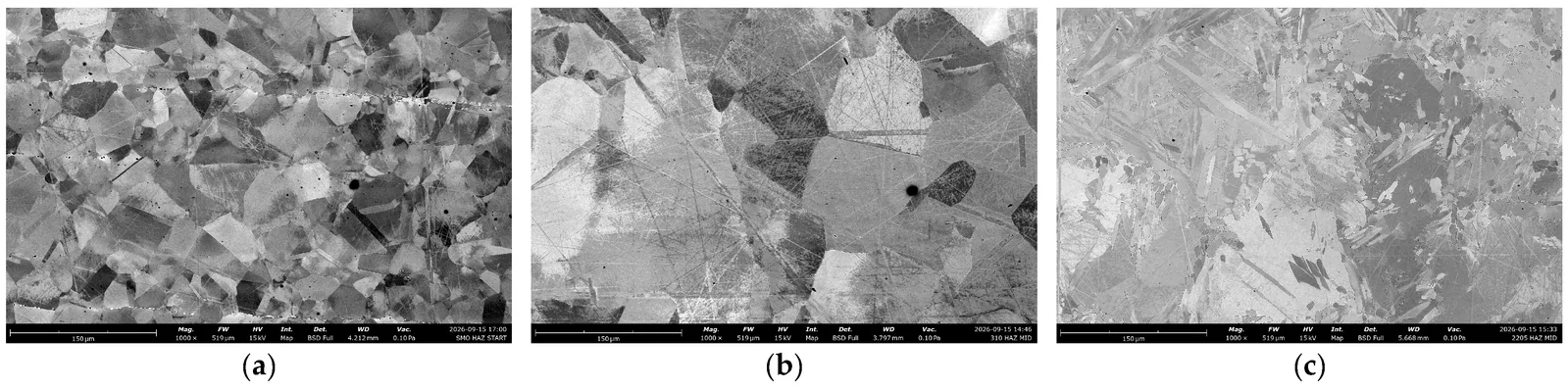
SEM micrographs of the HAZ: (**a**) 254 SMO, (**b**) 310S, and (**c**) 2205 duplex stainless steel.

Figure 6 shows representative HAZ microstructures for 254 SMO, 310S, and 2205. For each base material, the observed HAZ morphology was similar across the different dissimilar joints; therefore, one micrograph per material is presented and compared with the corresponding base metal in Figure 5. The 254 SMO HAZ retains the equiaxed austenitic structure and annealing twins observed in the base metal, although some grains appear larger than in the corresponding base-metal microstructure (Figure 5a and Figure 6a). Grain coarsening is more pronounced in the 310S HAZ, where the austenitic grains are visibly larger than those in the corresponding base metal, while annealing twins remain apparent (Figure 5b and Figure 6b). The 2205 HAZ exhibits a more substantial morphological change: the fine, approximately parallel ferrite–austenite bands of the base metal give way to larger ferritic regions and irregular austenitic features along the ferrite boundaries and within the ferritic regions (Figure 5c and Figure 6c). This morphology is consistent with ferrite coarsening during heating and austenite reformation during cooling. The ferrite–austenite phase fractions in the 2205 base metal and HAZ were not quantitatively determined; therefore, the observed microstructural changes are discussed qualitatively.

#### 3.2.3. Fusion-Boundary Microstructure and EDS Analysis

**Figure 7:**
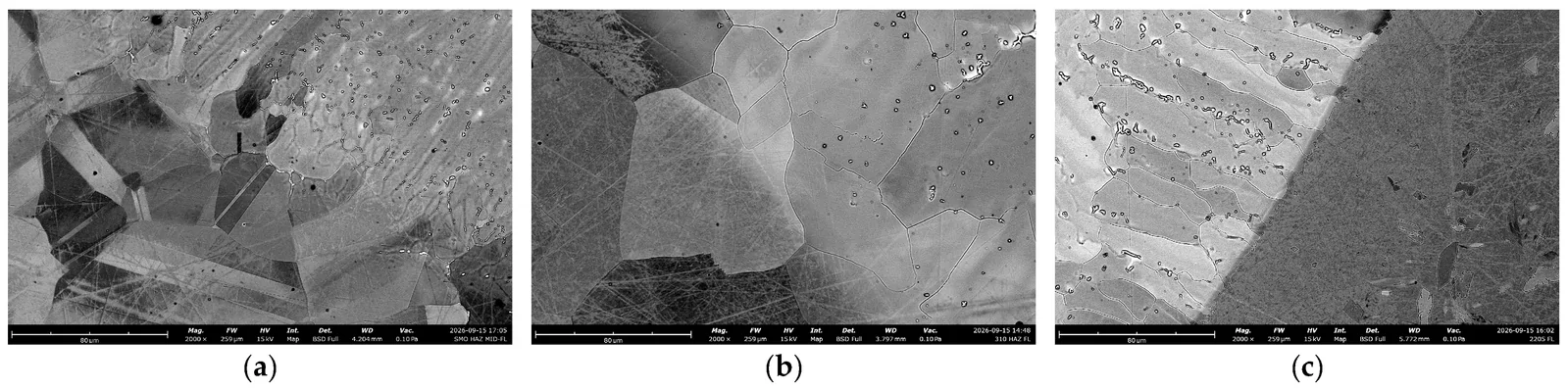
SEM micrographs of the fusion boundaries and adjacent heat-affected zones and weld metal: (**a**) 254 SMO, (**b**) 310S, and (**c**) 2205.

At the 254 SMO fusion boundary, twinned austenitic grains meet the weld metal along an irregular interface (Figure 7a). Bright-contrast constituents occur along some grain boundaries near the interface and are abundant in the adjacent weld metal. Their contrast and the composition of the ERNiCrMo-3 filler suggest Nb–Mo-enriched secondary phases. On the 310S side, the fusion boundary is locally diffuse, and annealing twins are not discernible in the austenitic region immediately adjacent to it (Figure 7b). The bright constituents are more sparsely distributed in this field and do not form a continuous network around the austenitic grain at the interface. The 2205 fusion boundary is clearly delineated and slightly serrated (Figure 7c). Elongated features in the adjacent weld metal are oriented away from the boundary, consistent with directional solidification, while bright constituents are also visible along some internal boundaries within the weld metal.

**Figure 8:**
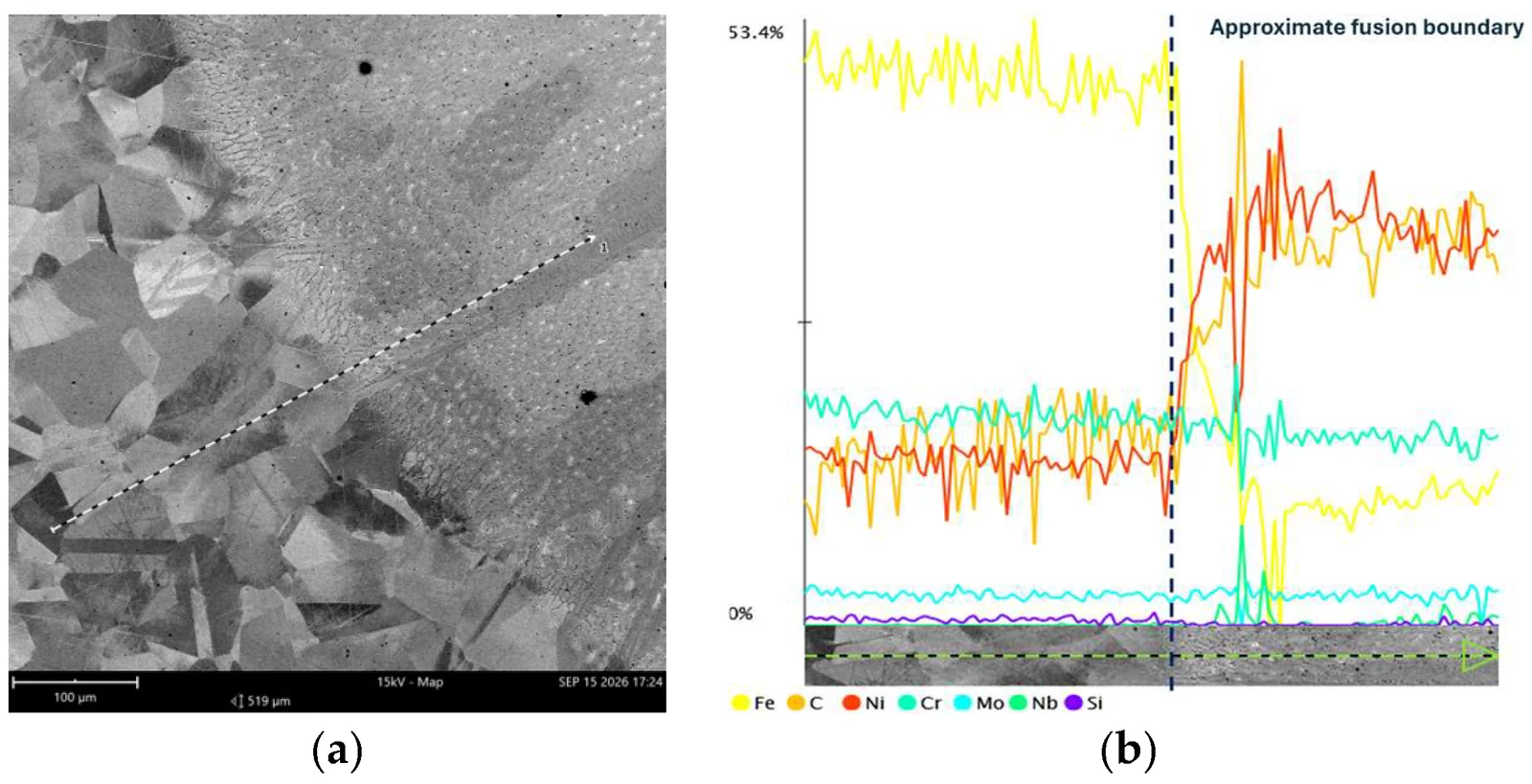
SEM micrograph and EDS line-scan profiles across the 254 SMO–ERNiCrMo-3 fusion boundary: (**a**) analysis path from 254 SMO to the weld metal; (**b**) corresponding elemental concentration profiles. The dashed line marks the approximate position of the fusion boundary.

The EDS line scan across the 254 SMO fusion boundary shows a marked decrease in Fe and an increase in Ni towards the ERNiCrMo-3 weld metal (Figure 8). The compositional change occurs over a relatively narrow section of the examined path. A sharp local Nb peak on the weld-metal side is consistent with the analysis line intersecting a discrete Nb-rich constituent.

**Figure 9:**
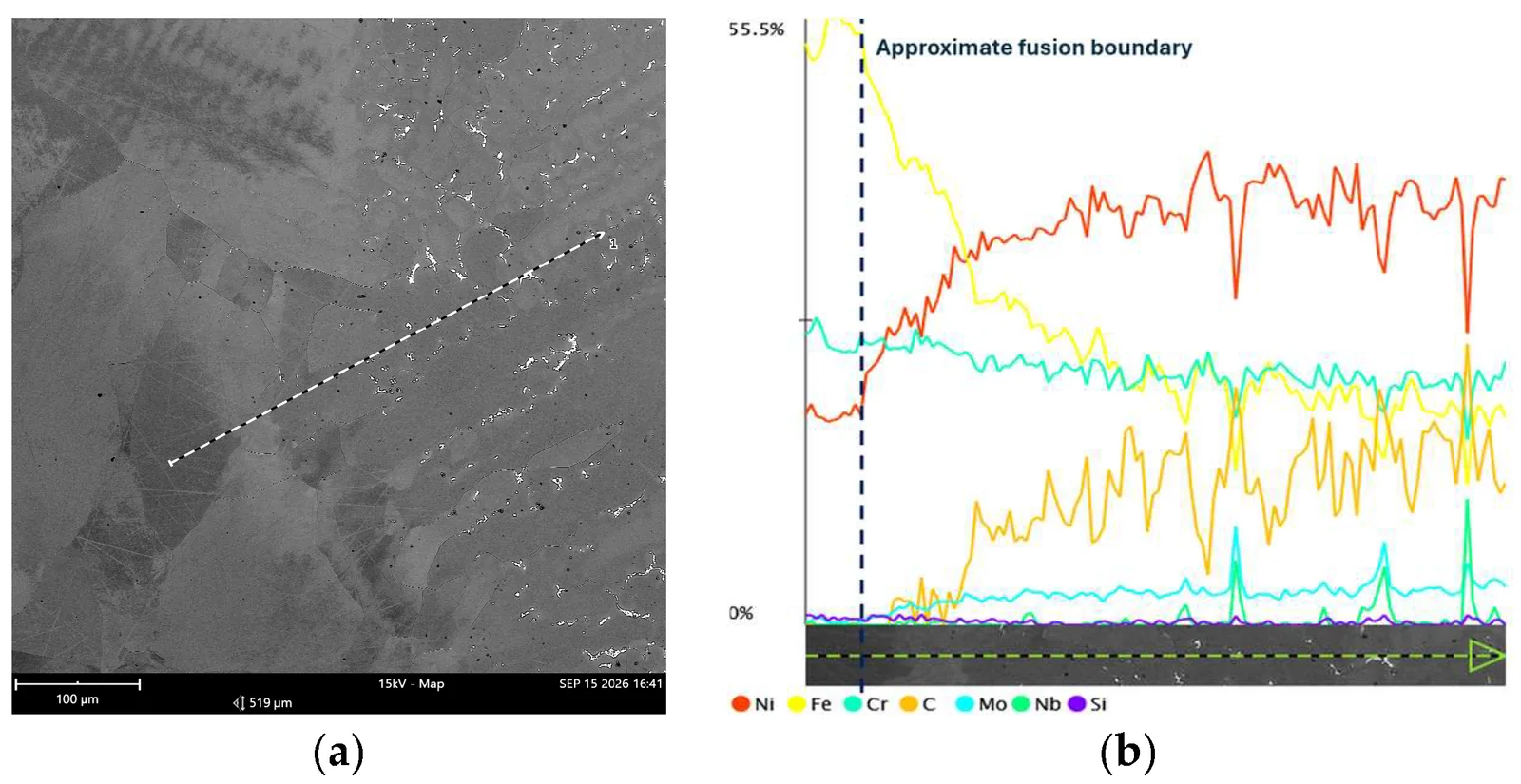
SEM micrograph and EDS line-scan profiles across the 310S–ERNiCrMo-3 fusion boundary: (**a**) analysis path from 310S to the weld metal; (**b**) corresponding elemental concentration profiles. The dashed line marks the approximate position of the fusion boundary.

Across the 310S fusion boundary, Fe decreases and Ni increases more gradually along the examined path (Figure 9). The broader compositional transition is consistent with local mixing of 310S and filler metal near the interface. Local Nb peaks occur within the weld metal, without evidence of a continuous Nb-rich interfacial layer.

**Figure 10:**
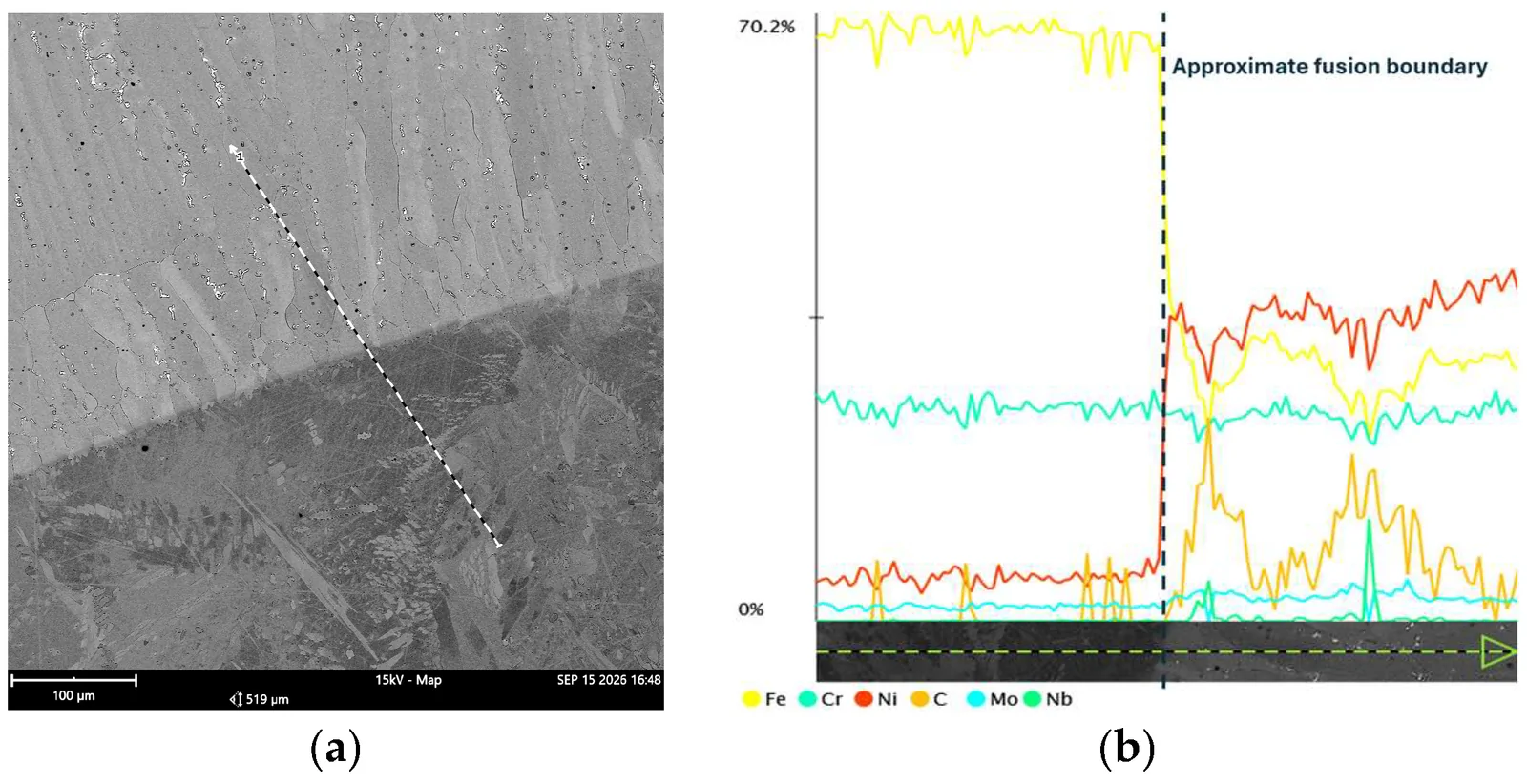
SEM micrograph and EDS line-scan profiles across the 2205–ERNiCrMo-3 fusion boundary: (**a**) analysis path from 2205 to the weld metal; (**b**) corresponding elemental concentration profiles. The dashed line marks the approximate position of the fusion boundary.

The 2205 line scan begins in the Fe-rich duplex base metal and shows a pronounced increase in Ni upon entering the ERNiCrMo-3 weld metal (Figure 10). Fe remains appreciable immediately beyond the fusion boundary, consistent with local dilution of the Ni-based weld metal by 2205. Narrow Nb peaks within the weld metal indicate discrete Nb-rich constituents along the analysis path.

The SEM–EDS line scans provide qualitative evidence of elemental redistribution across the fusion boundaries, consistent with local dilution of the Ni-based filler metal by the adjacent base metals. However, these measurements do not permit a reliable quantitative determination of the dilution fraction. Therefore, the effects of dilution on weld-metal chemistry and microstructural development are discussed qualitatively.

**Figure 11:**
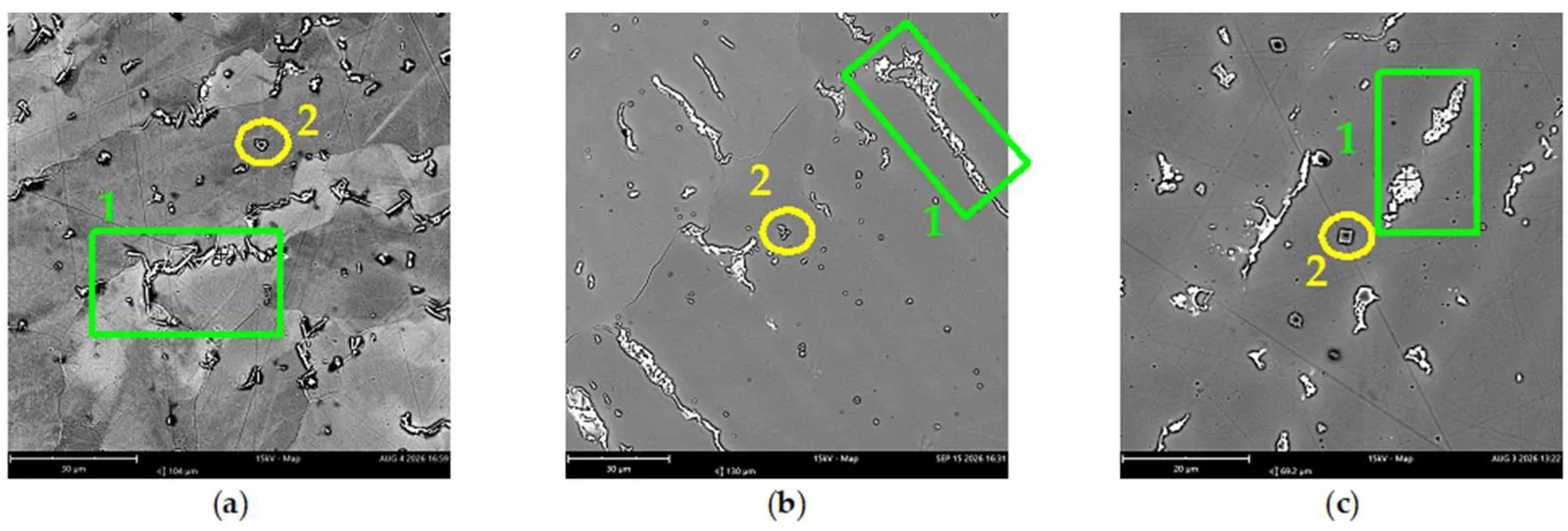
Backscattered-electron SEM images of secondary constituents in the ERNiCrMo-3 weld metal adjacent to the fusion boundaries with (**a**) 254 SMO, (**b**) 310S, and (**c**) 2205. Region 1 highlights bright, boundary-associated Nb–Mo-rich constituents; region 2 highlights a darker, geometrically shaped Ti–Nb-rich constituent. The corresponding EDS compositions are given in Table 3.

Two types of constituents were observed in the ERNiCrMo-3 weld metal adjacent to the fusion boundaries (Figure 11). The bright constituents in region 1 are distributed along dendrite boundaries in the weld metal. Adjacent to 254 SMO, they are slender and locally needle-like or branched; adjacent to 310S, they form longer interconnected segments; and adjacent to 2205, they appear as shorter, discontinuous features. These differences in morphology may be related to variations in local dilution and solidification conditions near each fusion boundary. Dilution from the different base metals can alter the composition of the residual interdendritic liquid and, consequently, the morphology and distribution of the constituents formed during solidification. EDS analysis shows Nb and Mo enrichment in the selected bright constituents (Table 3). The interdendritic distribution, morphology, and Nb–Mo enrichment of these constituents are consistent with potential Laves-type phases, as reported in Ni-based weld metals. Although their crystallographic structure was not directly determined, the combined morphological and compositional observations support this metallurgical interpretation. The locally elevated carbon content (4.76 wt.%) detected in the Nb–Mo-rich constituent adjacent to 2205 also suggests the possible presence of a carbide-containing constituent. The darker constituents in region 2 are discrete and angular, in places approximately cuboidal, and show pronounced Ti and Nb enrichment. Their distinct morphology and composition are consistent with Ti–Nb-rich particles within the weld metal.

#### 3.2.4. Weld-Metal Center

**Figure 12:**
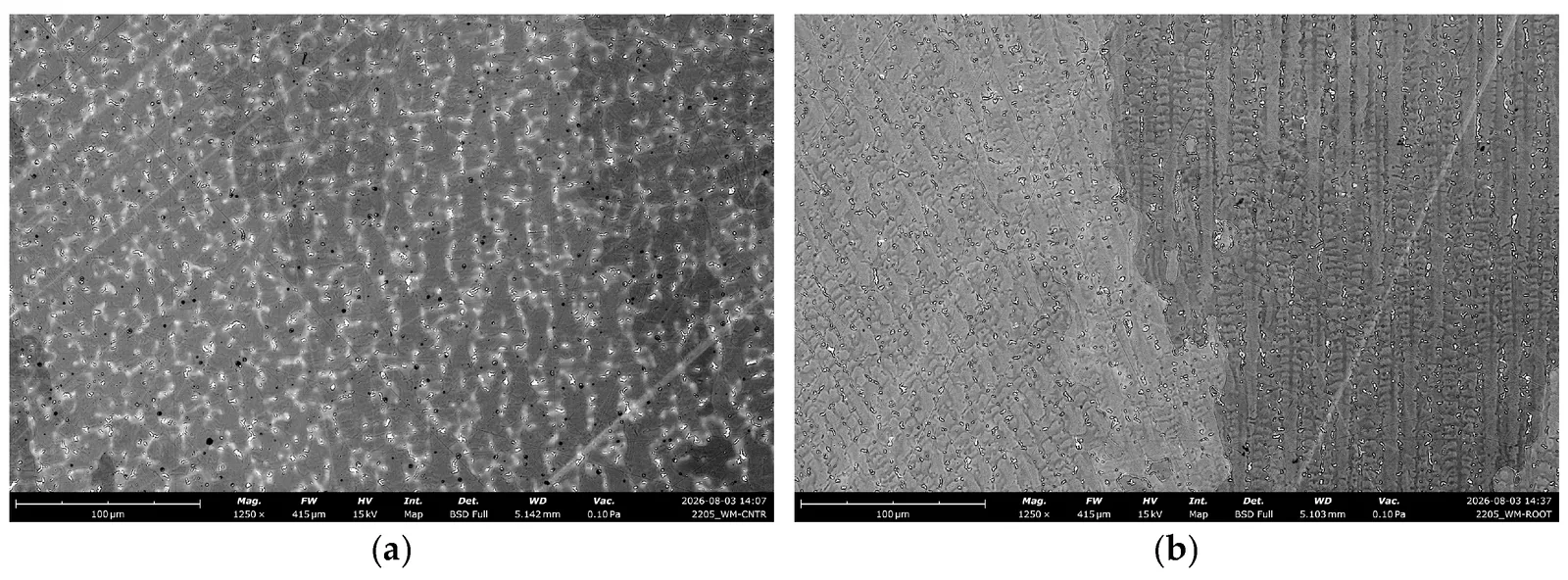
SEM micrographs of the ERNiCrMo-3 weld metal showing (**a**) an equiaxed-appearing cellular–dendritic structure near the center of a pass and (**b**) elongated, directionally aligned dendritic features near the boundary between successive passes.

The changes in solidification-feature orientation identified in the stereomicrographs of the three joints (Figure 2, Figure 3 and Figure 4) are more clearly resolved by SEM (Figure 12). Near the center of a weld pass, the structure has a relatively equiaxed, cellular–dendritic appearance, with no dominant growth direction across the examined field (Figure 12a). Closer to the boundary between successive passes, elongated dendritic features become directionally aligned (Figure 12b). Their orientation is consistent with growth along the local thermal gradient as heat was extracted through the previously deposited pass. The transition from directional to more equiaxed-appearing growth is therefore associated with the position within the weld pool and the local solidification geometry. Bright constituents are distributed along the interdendritic boundaries in both fields. Their location is consistent with solute partitioning into the residual liquid during weld-metal solidification and their distribution resembles that observed near the fusion lines (Figure 11). The same broad combination of directional and equiaxed-appearing solidification features was observed in the weld metal of all three dissimilar joints.

### 3.3. Microhardness Analysis

**Figure 13:**
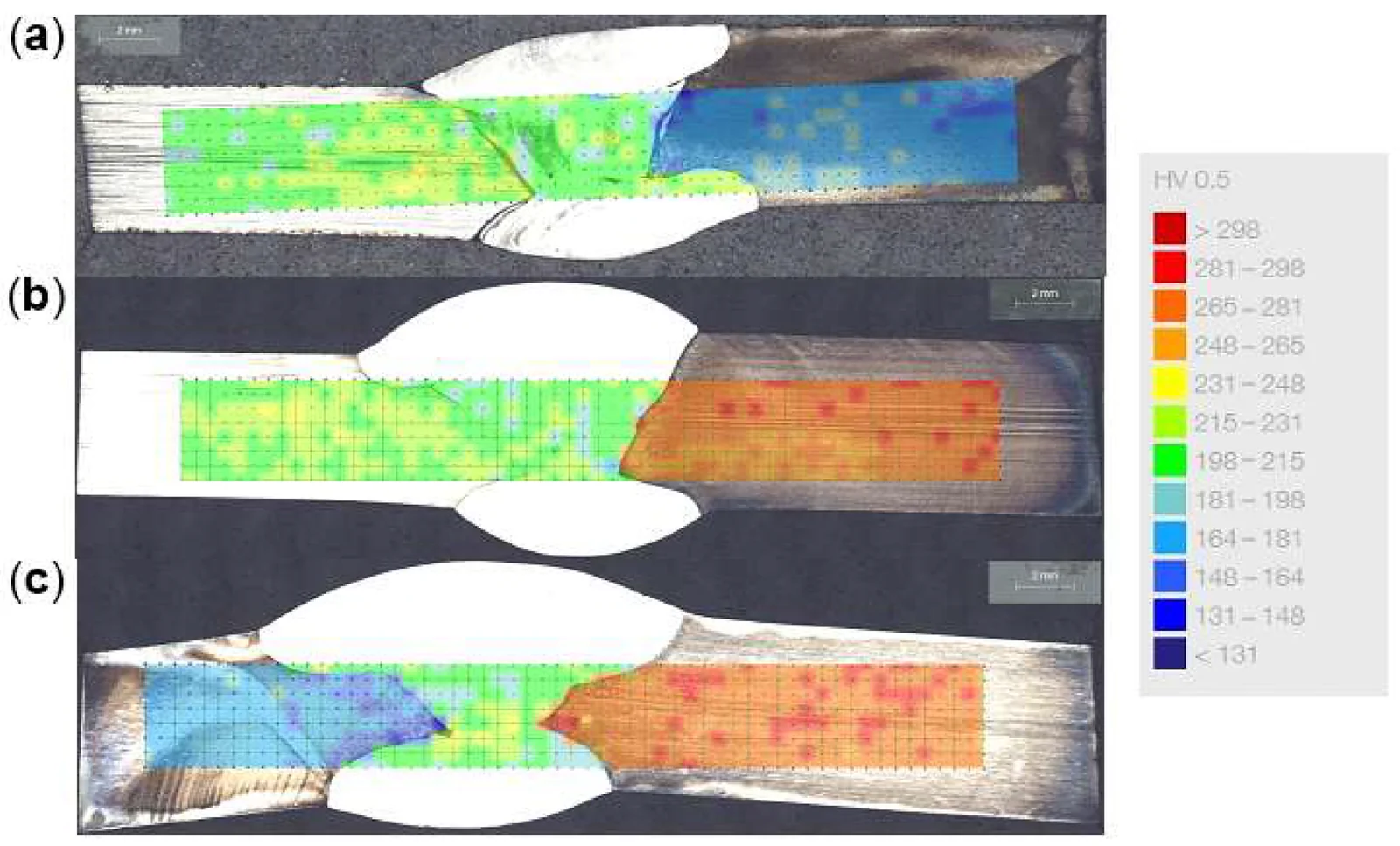
Vickers microhardness maps (HV0.5) superimposed on transverse cross-sections of the (**a**) 254 SMO–310S, (**b**) 310S–2205, and (**c**) 254 SMO–2205 welded joints. The color scale indicates the measured hardness in HV0.5.

**Figure 14:**
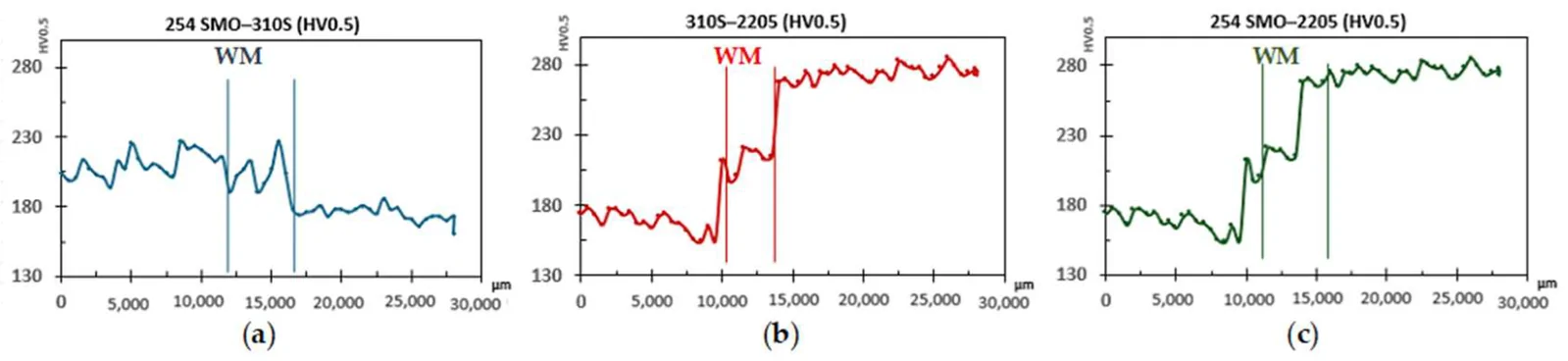
Vickers microhardness (HV0.5) profiles across the dissimilar welded joints: (**a**) 254 SMO–310S, (**b**) 310S–2205, and (**c**) 254 SMO–2205, measured along the mid-thickness line at 500 μm intervals. The region between the vertical lines corresponds to the weld metal.

The microhardness maps and mid-thickness profiles presented in Figure 13 and Figure 14, respectively, reveal distinct hardness distributions among the three dissimilar joints. The 254 SMO and 310S base metals exhibit comparatively similar hardness levels, consistent with their fully austenitic microstructures, whereas substantially higher values are associated with the 2205 side. This difference is also evident in the 310S–2205 and 254 SMO–2205 joints, where a pronounced hardness transition occurs from the austenitic or super-austenitic base metal towards the weld metal and duplex side. The differences in the measured hardness of the 254 SMO base metal between the two joints may be associated with local measurement variability and, to some extent, differences in welding heat input and the resulting thermal exposure. However, these variations do not indicate a substantial change in the overall hardness characteristics of 254 SMO.

Although the same ERNiCrMo-3 filler metal was used for all three joints, the weld-metal hardness was not identical, as shown by the profiles in Figure 14. This behavior is consistent with the different dilution conditions imposed by the adjoining base metals. The EDS line scans showed progressive changes in Fe and Ni across the fusion boundaries and appreciable Fe within the weld metal adjacent to 2205, consistent with local dilution of the Ni-based filler. In addition, the weld metal contained interdendritic Nb–Mo-rich constituents and discrete Ti–Nb-rich particles, whose morphology varied with the adjacent base metal. These compositional and solidification-related heterogeneities may contribute to the observed spatial variation in hardness. No narrow region of extreme hardness is evident at the fusion boundaries. The hardness changes are predominantly gradual or step-like across the transition between the base metals and weld metal. This observation is consistent with the SEM–EDS results, which show that the Nb- and Mo-enriched constituents occur as discrete interdendritic features rather than as a continuous interfacial layer. The observed hardness distributions are therefore consistent with differences in base-metal phase constitution, weld-metal dilution, and solidification-induced chemical heterogeneity.

### 3.4. Mechanical Properties

**Figure 15:**
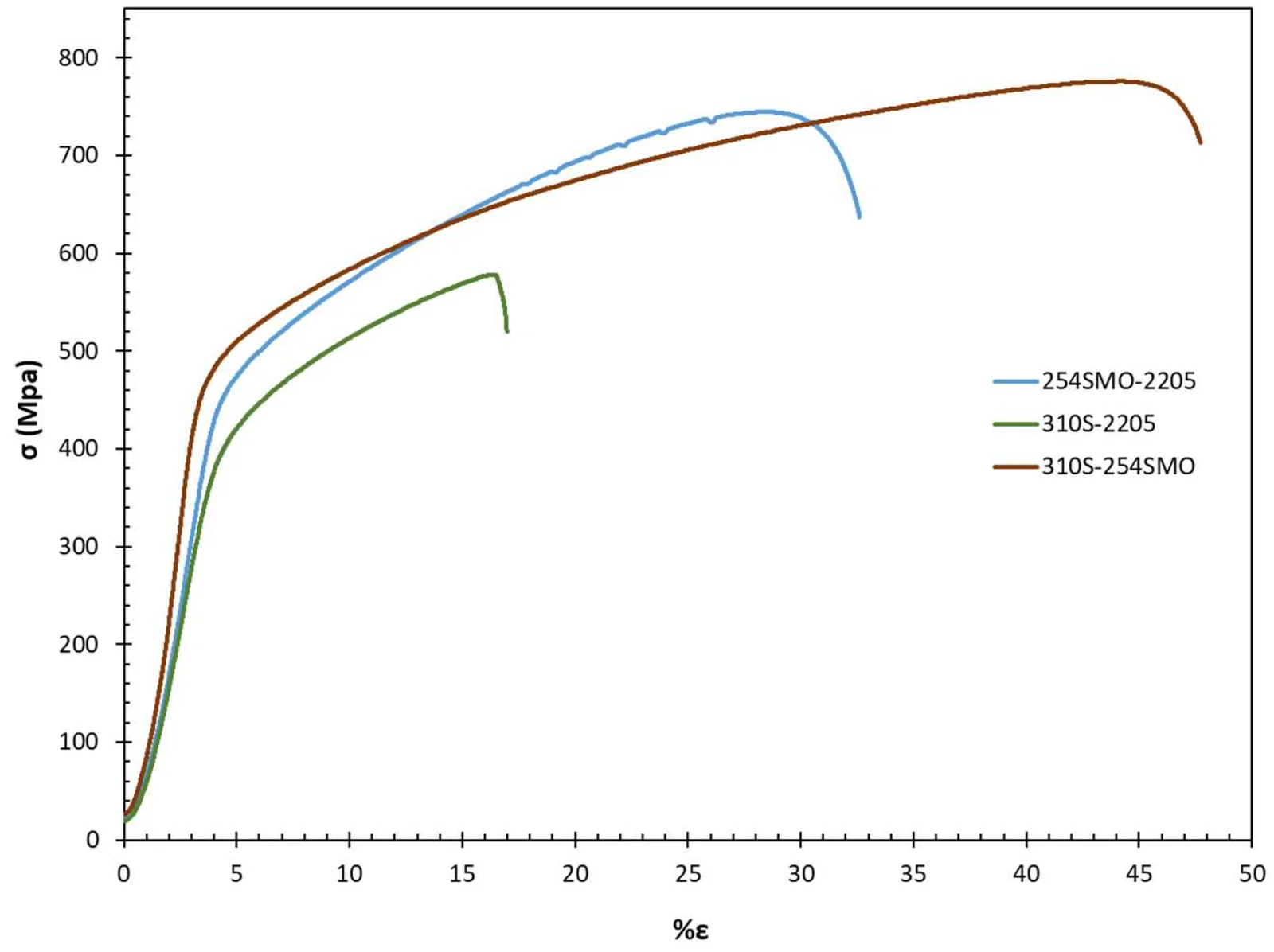
Engineering stress–strain curves obtained from transverse tensile tests of the three dissimilar welded joints.

The engineering stress–strain curves obtained from the transverse tensile tests are presented in Figure 15, while the corresponding mechanical properties are summarized in Table 4. The three dissimilar joints exhibited markedly different tensile responses. The 254 SMO–310S joint showed the highest ultimate tensile strength (776 MPa), 0.2% proof strength (463 MPa), and elongation (43.6%). The 254 SMO–2205 joint exhibited slightly lower strength, with an ultimate tensile strength of 742 MPa and a 0.2% proof strength of 447 MPa, while retaining a comparatively high elongation of 27.7%. In contrast, the 310S–2205 joint exhibited the lowest ultimate tensile strength (578 MPa), 0.2% proof strength (388 MPa), and elongation (12.3%), indicating a substantially reduced deformation capacity compared with the other two dissimilar joints.

**Figure 16:**
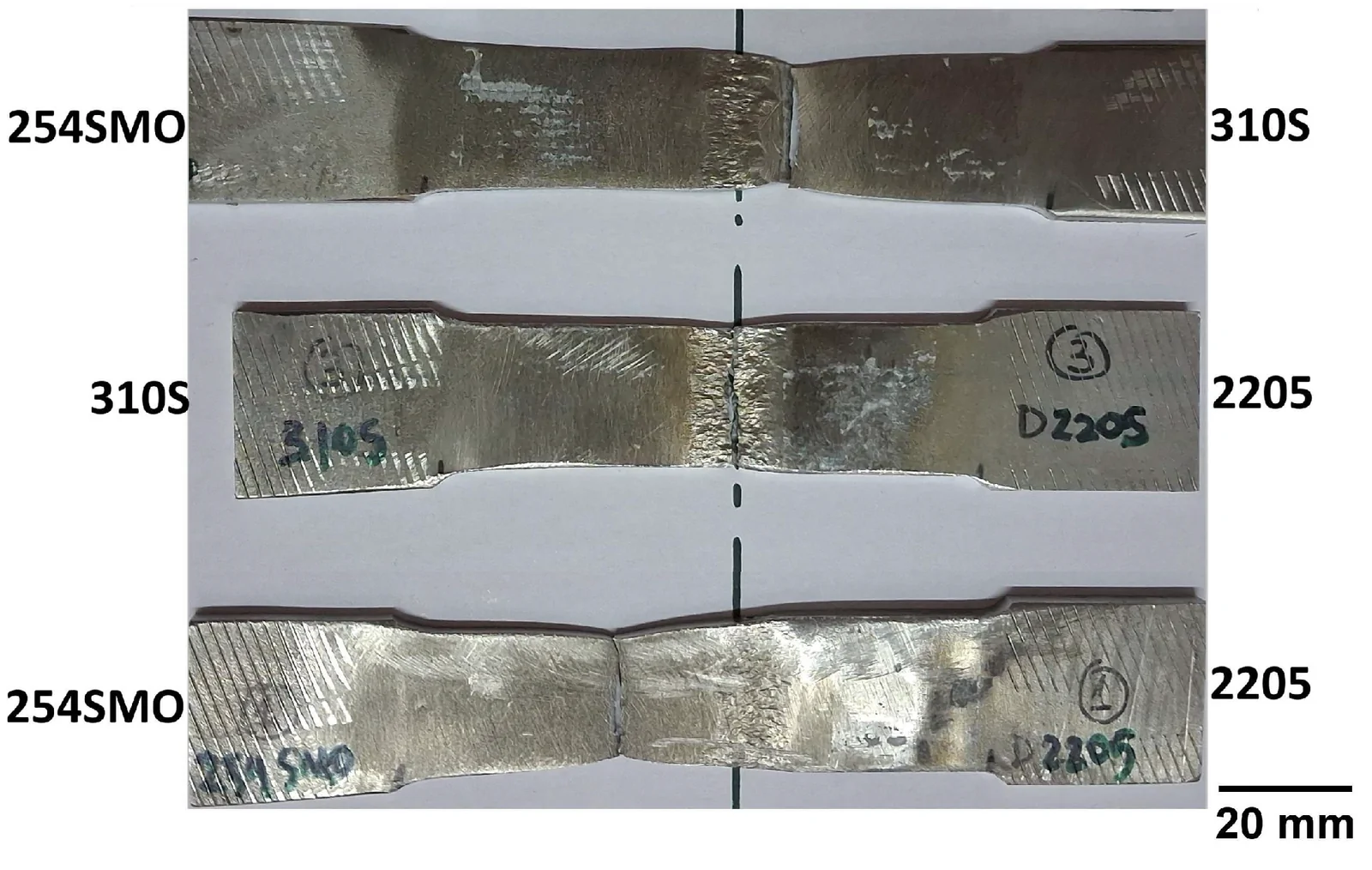
Tensile specimens after uniaxial testing, showing the fracture locations of the 254 SMO–310S, 310S–2205, and 254 SMO–2205 dissimilar welded joints. The dashed line indicates the approximate weld centerline.

Comparison with the indicative mechanical properties in Table 4 shows that the 254 SMO–310S and 254 SMO–2205 joints achieved tensile strengths exceeding the reference values of their constituent base metals, with fracture occurring outside the welded region (Figure 16). Conversely, the 310S–2205 joint fractured within the welded region at 578 MPa, below the indicative strengths of 2205 (655 MPa) and ERNiCrMo-3 deposited weld metal (740 MPa), but above that of 310S (515 MPa). All specimens exhibited macroscopically ductile fracture characteristics, without evidence of macroscopic welding discontinuities. The lower tensile performance of the 310S–2205 joint may reflect local variations in the mechanical response of the welded region, although no specific failure mechanism was established.

The fracture locations after tensile testing are shown in Figure 16 and provide additional insight into the different mechanical responses. Fracture of the 254 SMO–310S joint occurred on the 254 SMO side, whereas the 254 SMO–2205 specimen failed on the 2205 side. In both cases, fracture occurred outside the weld zone, indicating that the ERNiCrMo-3 weld metal and the fusion regions were able to sustain the applied tensile load without becoming the preferential sites of failure. In contrast, the 310S–2205 specimen fractured within the weld zone, coinciding with its substantially lower tensile strength and elongation. For the tested 310S–2205 specimen, the weld zone was therefore the preferential fracture location.

These differences in tensile behavior are consistent with the microstructural and microhardness results presented in Section 3.2 and Section 3.3. The 254 SMO–310S joint exhibited the smoothest hardness distribution across the joint, with the fully austenitic base metals and weld region showing relatively moderate hardness differences. Its high elongation and fracture outside the weld zone indicate that the compositional and microstructural heterogeneity introduced by welding did not produce a mechanically dominant weak region under transverse tensile loading.

The behavior of the 254 SMO–2205 joint was different because of the pronounced mechanical mismatch between the super-austenitic and duplex sides. The microhardness profile showed a substantial increase in hardness towards the 2205 side, consistent with its ferritic–austenitic phase constitution and with the welding-induced microstructural changes observed in the duplex HAZ. Nevertheless, fracture occurred on the 2205 side rather than within the ERNiCrMo-3 weld metal. The combination of relatively high tensile strength and appreciable elongation, together with failure outside the weld zone, indicates that the welded region of the tested specimen retained adequate tensile integrity despite the strong hardness gradient across the joint.

The 310S–2205 joint exhibited a distinctly different response. Its lower elongation and tensile strength, together with fracture within the weld zone, indicate that failure occurred within the heterogeneous welded region. This joint combines the fully austenitic 310S with the ferritic–austenitic 2205 duplex stainless steel and therefore presents a pronounced difference in phase constitution and mechanical response across the joint. The SEM–EDS examination further showed compositional gradients associated with dilution of the Ni-based ERNiCrMo-3 weld metal and the presence of interdendritic Nb–Mo-rich constituents and discrete Ti–Nb-rich particles. Such microstructural and compositional heterogeneity, together with the local hardness gradients observed across the joint, may contribute to strain localization and the reduced deformation capacity of this material combination.

Overall, the tensile results demonstrate that the mechanical performance of the dissimilar joints cannot be interpreted solely from the nominal strength of the constituent base metals or filler metal. Rather, the fracture behavior reflects the combined influence of base-metal phase constitution, local mechanical mismatch, weld-metal dilution, solidification-induced segregation, and the resulting spatial variation in hardness. The failure of the 254 SMO–310S and 254 SMO–2205 specimens outside the weld zone indicates adequate tensile integrity of their welded regions under the applied loading conditions, whereas the 310S–2205 specimen fractured within the weld zone and exhibited the lowest tensile strength and elongation of the three material combinations.

### 3.5. Corrosion Resistance

The specimens showed limited localized corrosion under the adopted ASTM G48 [34] test conditions. No pronounced or extensive pitting was observed, although a limited number of very small localized surface features were detected. This observation was consistent with the low measured mass losses summarized in Table 5.

The corrosion assessment was based on mass-loss measurements and visual examination of the exposed surfaces. Although no pronounced pitting or preferential attack of the welded regions was observed, the presence of very small localized pits cannot be excluded. No quantitative pit-depth or pit-density measurements were performed. The measured mass loss increased in the order 254 SMO–2205 < 254 SMO–310S < 310S–2205, indicating differences in corrosion response among the three joints under the applied test conditions. No significant preferential attack was observed in the base metal (BM), heat-affected zone (HAZ), fusion zone (FZ), or along the fusion boundaries, indicating that neither the weld metal nor the HAZ acted as a preferential site for localized corrosion initiation under the conditions investigated. This is particularly relevant to the dissimilar joints, where differences in chemical composition, phase constitution, dilution, and thermal history may produce local variations in corrosion resistance. The observed behaviour can be qualitatively related to the Pitting Resistance Equivalent Number (PREN) of the constituent materials. The nominal PREN values were calculated using the formula PREN = Cr + 3.3Mo + 16N, where Cr, Mo, and N are expressed in wt.%. These values refer to the nominal compositions of the constituent materials and should not be interpreted as experimentally determined PREN values of the diluted weld metal. The calculated PREN values for 310S, 2205, 254SMO, and the ERNiCrMo-3 filler metal were 25.00, 35.95, 43.83, and 51.20, respectively. The relatively low mass loss of the 254SMO-containing joints is consistent with the high Cr–Mo–N content and corresponding PREN of 254SMO, whereas the higher mass loss of the 310S–2205 joint may be partly associated with the lower pitting resistance of 310S. Within the joints investigated, the presence of 310S was therefore associated with comparatively greater susceptibility to localized corrosion. The absence of significant attack in the FZ suggests that the selected ERNiCrMo-3 filler metal and welding conditions provided adequate resistance to pitting under the applied test conditions. Nevertheless, the corrosion behaviour of the weld metal may be affected by dilution from the two base metals, elemental segregation during solidification, phase constitution, and local Cr, Mo, and N distributions. Therefore, the observed corrosion behaviour should be interpreted based on the actual weld-metal composition and microstructure rather than solely on the nominal PREN values of the constituent materials. Overall, all three dissimilar joints demonstrated high resistance to pitting initiation, with very low mass losses and no significant preferential corrosion of the BM, HAZ, or FZ.

## 4. Discussion

The present results show that the response of the investigated dissimilar stainless-steel joints was strongly influenced by the interaction between the ERNiCrMo-3 filler metal and the different chemical compositions and phase constitutions of the adjoining base metals. Although the same filler metal and welding process were used, the three material combinations developed different microstructural, mechanical, and corrosion responses. These differences emphasize the importance of considering the combined base-metal/filler-metal system when evaluating dissimilar stainless-steel welds.

The welding thermal cycle produced material-dependent HAZ transformations. The austenitic 254 SMO and 310S largely retained their original phase constitution, although grain coarsening was observed, particularly in 310S, whereas the 2205 HAZ underwent a more pronounced modification of its ferritic–austenitic morphology. This behavior is consistent with previous observations of microstructural changes in the HAZ of austenitic/duplex dissimilar welds [15,16,17,18]. Within the weld metal, the Fe–Ni gradients measured across the fusion boundaries demonstrated dilution of the Ni-based filler by the adjoining base metals. At the same time, Nb–Mo-rich interdendritic constituents and discrete Ti–Nb-rich particles were observed in the weld metal. Their different morphology adjacent to the three base metals suggests that local dilution influenced solute partitioning and the resulting weld-metal heterogeneity. The Nb–Mo-rich constituents were morphologically and compositionally consistent with Laves-type constituents. Similar Nb- and Mo-rich segregation has been reported in dissimilar stainless-steel welds produced with ERNiCrMo-3 filler metal [14,19], while Laves-phase formation has been specifically reported for the AL6XN/2205 combination [14].

These metallurgical differences were reflected in the hardness and tensile behavior. Although only one tensile specimen was tested per welded combination, the results were evaluated alongside extensive metallographic, SEM–EDS, and microhardness characterization. Therefore, the tensile measurements provide complementary information on the mechanical behavior of the investigated joints, although additional tests would be required to establish statistical repeatability. Since one tensile specimen was tested for each dissimilar material combination, the reported mechanical properties should be interpreted as individual experimental results, without a statistical assessment of repeatability.

The 254 SMO–310S joint exhibited the smoothest hardness transition and combined the highest tensile strength (776 MPa) with the highest elongation (43.6%), fracturing outside the weld zone. The 254 SMO–2205 joint exhibited an ultimate tensile strength of 742 MPa despite the pronounced hardness mismatch associated with the duplex side and also fractured outside the weld zone. In contrast, the 310S–2205 joint exhibited substantially lower tensile strength and elongation (578 MPa and 12.3%, respectively) and fractured within the weld zone. These differences suggest that the tensile response was influenced by the combined effects of dilution, solidification-induced heterogeneity, phase constitution, and local mechanical mismatch rather than by the nominal strength of the filler metal alone. This interpretation is consistent with previous studies of austenitic/duplex dissimilar welds, in which filler-metal selection and the resulting weld microstructure were shown to influence mechanical behavior [19,20].

Tensile testing was performed on one specimen per dissimilar welded combination, and therefore the measured mechanical properties cannot be considered statistically representative. Furthermore, minor variations in heat input were inherent to the manual GTAW process, while the investigation was limited to the selected welding conditions and material combinations. Nevertheless, the combined metallographic, SEM–EDS, microhardness, and mechanical characterization provides a comprehensive basis for interpreting the observed behavior of the investigated joints.

The corrosion results complement this interpretation. All three joints showed low mass loss and no pronounced preferential attack of the weld metal, HAZ, or fusion boundaries under the applied ASTM G48 conditions, indicating that the weld metal did not constitute a preferential site for localized corrosion under the conditions investigated. Nevertheless, the increase in mass loss from 254 SMO–2205 to 254 SMO–310S and 310S–2205 indicates that the base-metal combination influenced the corrosion response of the joints. This dependence on local composition and microstructure is consistent with previous studies reporting variations in corrosion behavior with weld microstructure, welding conditions, and filler-metal selection in dissimilar stainless-steel joints [11,12,22]. Taken together, the results indicate that the performance of ERNiCrMo-3 dissimilar stainless-steel welds depends on the interaction between filler-metal composition, dilution, phase constitution, and the specific combination of adjoining base metals.

## 5. Conclusions

Dissimilar 254 SMO–310S, 254 SMO–2205, and 310S–2205 joints were produced by GTAW using ERNiCrMo-3 filler metal. All three joints exhibited full penetration without visible macroscopic discontinuities in the examined cross-sections. Despite the use of the same filler metal and welding procedure, the three material combinations developed different microstructural and mechanical responses.

SEM–EDS analysis revealed compositional gradients consistent with base-metal-dependent dilution of the Ni-based weld metal, together with solidification-induced chemical heterogeneity, including Nb–Mo-rich interdendritic constituents and discrete Ti–Nb-rich particles. These differences were reflected in the mechanical response: the 254 SMO–310S and 254 SMO–2205 specimens fractured outside the weld zone, whereas the 310S–2205 specimen fractured within the weld and exhibited the lowest tensile strength and elongation.

Under the applied ASTM G48 conditions, all three joints showed low mass loss and no pronounced preferential attack of the weld metal, HAZ, or fusion boundaries. The results show that the response of ERNiCrMo-3 dissimilar welds depends on the specific combination of adjoining base metals, through differences in dilution, phase constitution, weld-metal heterogeneity, and local mechanical mismatch.

## Figures and Tables

**Table 1:** Nominal chemical compositions of the base metals and filler metal (wt.%).

Material	C	Mn	P	S	Si	Cr	Ni	Mo	N	Cu	Fe	Other Reported Elements
310S (UNS S31008)	0.08	2.00	0.045	0.030	1.50	24.0–26.0	19.0–22.0	—	—	—	Bal.	—
254 SMO (UNS S31254)	0.020	1.00	0.030	0.010	0.80	19.5–20.5	17.5–18.5	6.0–6.5	0.18–0.22	0.50–1.00	Bal.	—
2205 (UNS S32205)	0.03	2.00	0.030	0.020	1.00	22.0–23.0	4.5–6.5	3.0–3.5	0.14–0.20	—	Bal.	—
ERNiCrMo-3	0.10	0.50	0.020	0.015	0.50	20.0–23.0	≥58.0	8.0–10.0	—	0.50	5.0	Nb + Ta: 3.15–4.15; Al: ≤0.40; Ti: ≤0.40

**Table 2:** Welding parameters used for the dissimilar joints.

Welded Joint	Pass ^1^	Current (A)	Voltage (V)	Travel Speed (mm/min)	Heat Input (kJ/mm)
254 SMO–310S	F1	70	12	28	1.08
	F2	75	12	30	1.08
	R1	80	14	35	1.15
254 SMO–2205	F1	80	12	30	1.15
	F2	85	12.5	32	1.20
	R1	90	12	30	1.30
310S–2205	F1	80	11.5	28	1.18
	F2	85	12	30	1.22
	R1	90	12.5	32	1.27

^1^ F1 and F2 denote passes deposited from the face side; R1 denotes the pass deposited from the reverse side (root side).

**Table 3:** EDS compositions of the constituents identified as regions 1 and 2 in Figure 11 (wt.%).

Material	EDS Spot No.	Element %wt								
		C	Fe	Cr	Ni	Mo	Nb	Ti	Si	P
254 SMO	1	-	11.27	21.42	8.51	17.31	41.49	-	-	-
	2	-		17.16	-	7.72	39.11	36.01	-	-
310S	1	-	13.80	16.59	30.40	18.06	19.66	-	0.88	0.61
	2	-	-	15.81	-	8.28	35.83	40.08	-	-
2205	1	4.76	12.18	15.53	29.61	21.94	14.75	-	0.68	0.55
	2	-	-	5.73	-	5.48	45.63	43.16	-	-

**Table 4:** Reference mechanical properties of the base metals and ERNiCrMo-3 filler metal, compared with the experimental tensile properties of the dissimilar welded joints.

	Specimen	0.2% Proof Strength, Rp0.2 (MPa)	Ultimate Tensile Strength, UTS (MPa)	Elongation (%)
Reference values	254SMO	320.0	680.0	50.0
	310S	205.0	515.0	40.0
	2205	450.0	655.0	25.0
	ERNiCrMo-3	460.0	740.0	30.0
Experimental results	254SMO-2205	447.0	742.0	27.7
	310S-2205	388.0	578.0	12.3
	254SMO-310S	463.0	776.0	43.6

**Table 5:** Mass loss measured after the ASTM G48 pitting corrosion test.

Specimen	Δm (mg)	Δm/A (mg/cm^2^)	Δm (%)
254SMO-2205	0.0	0.00	0.00
310S-2205	31.8	0.93	0.05
254SMO-310S	4.8	0.15	0.01

## Data Availability

The original contributions presented in this study are included in the article. Further inquiries can be directed to the corresponding author.
